# Effective treatment of 2,4,6-trinitrotoluene from aqueous media using a sono–photo-Fenton-like process with a zero-valent iron nanoparticle (nZVI) catalyst†

**DOI:** 10.1039/d4ra03907f

**Published:** 2024-07-29

**Authors:** Hoang Van Nguyen, Son Tung Pham, Toan Ngoc Vu, Huong Van Nguyen, Duong Duc La

**Affiliations:** a Institute of New Technology Hanoi Vietnam sontung231@gmail.com; b Institute of Chemistry and Materials Hanoi Vietnam duc.duong.la@gmail.com

## Abstract

In this study, we examine the effectiveness of using a combination of a sono–photo-Fenton-like procedure and nano zero-valent iron catalyst (nZVI) to treat 2,4,6-trinitrotoluene (TNT) in an aquatic environment. Zero-valent iron particles were generated by a chemical reduction technique. nZVI nanoparticles were analyzed using scanning electron microscopy (SEM) and X-ray diffraction (XRD) to characterize the nanocatalyst. The resulting nZVI nanoparticles were used as an addition in a sono–photo-Fenton method to remediate an aqueous solution contaminated with TNT. Furthermore, influences of operational factors such as temperature, catalyst dosage, wavelength, ultraviolet power, ultrasonic frequency and power, pH level, H_2_O_2_/nZVI ratio, initial TNT concentration, and reaction duration on the treatment of TNT were investigated. Under the conditions of an ideal pH of 3, temperature range of 40–45 °C, concentration of 50 mg per L TNT, dose of 2 mM of nZVI, and ratio of H_2_O_2_/Fe^0^ of 20, a treatment efficiency of 95.2% was achieved after a duration of 30 minutes. The sono–photo-Fenton process combined with nZVI showed a higher TNT removal efficiency compared to the Fenton, sono-Fenton, and photo-Fenton processes under the same conditions. Moreover, it promises a potential solution to treat TNT at the pilot scale while allowing reuse of the nZVI catalyst and the limitation of sludge.

## Abstract 2

This work provides an effective approach to remove 2,4,6-trinitrotoluene from aqueous media using a sono–photo-Fenton-like process with catalytic zero-valent iron nanoparticles (nZVI).

## Graphical abstract

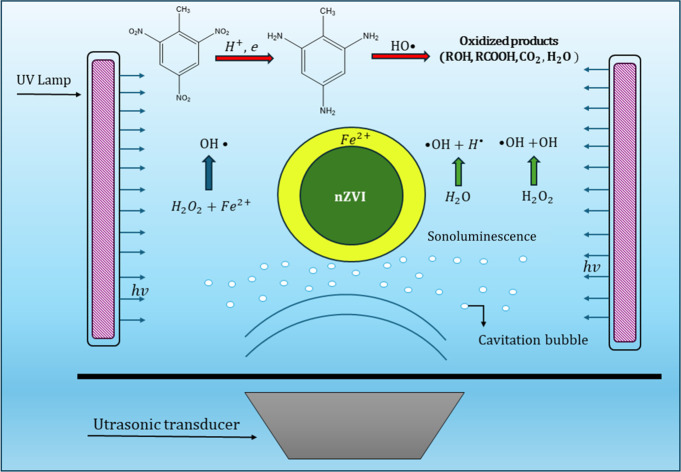

## Introduction

TNT, also known as 2,4,6-trinitrotoluene, is a highly used explosive in both military and industrial settings. During the production and use of TNT, wastewater is generated that contains various persistent organic compounds, particularly nitrotoluene group compounds, including mononitrotoluene (MNT) and 2,4-dinitrotoluene (DNT). These compounds can contribute to increased pollution in soil and groundwater, posing risks to human health as well as the well-being of fish, algae, earthworms, and microbes.^1–4^ Water contamination resulting from TNT can result in several ailments, such as allergies, liver damage, a compromised immune system, skin irritation, decreased appetite, anemia, and cancer.^5^

The sono–photo-Fenton-like process has gained significant attention recently because of its ability to efficiently degrade pollutants while also being energy- and cost-efficient.^6–10^ This process is a combination of a Fenton-like process with ultrasonic (US), ultraviolet radiation (UV), and heterogeneous catalysis. Under UV irradiation, Fe(OH)^2+^ complexes decompose to produce Fe^2+^ ion and OH˙. Meanwhile, under the impact of ultrasonic waves with high intensity, the explosion of cavitation bubbles creates a reaction area with a pressure of 500 atm, and temperature of 5200 K and 1900 K in the surrounding area.^11–14^ Under these conditions, several radicals such as OH˙, H˙, OOH˙, and O˙ are formed by the decomposition of water. In addition, the sonoluminescence phenomenon causes the decomposition of FeOOH^2+^ complex.^5^ The mechanism of the sono–photo-Fenton process is described by eqn (1)–(6) below.

Fenton reaction:

1 Fe^2+^ + H_2_O_2_ + H^+^ → Fe^3+^ + OH˙+ H_2_O

Under ultraviolet irradiation (UV):

2 Fe^3+^ + H_2_O → Fe(OH)^2+^ + OH^+^

3 Fe(OH)^2+^ ⇄ Fe^3+^ + OH^−^

4 Fe(OH)^2+^ + *hν* → Fe^2+^ + OH˙

Under ultrasonic treatment (US):

5 H_2_O → H˙ + OH˙

6 FeOOH^2+^ + ultrasound → Fe^2+^ + ˙HO_2_

Heterogeneous catalysts in the Fenton-like process have advantages such as reducing the amount of sludge, the ability to recover and reuse, increasing the amount of free hydroxyl formed on the catalyst surface, use in a wider pH range, and ability to treat wastewater containing high concentrations of pollutants.^15,16^ One of the common heterogeneous catalysts is nano zero-valent iron (nZVI). In the Fenton-like process, zero-valent nano iron can perform roles such as a reducing agent in direct reaction with nitro pollutants (–NO_2_) and a renewable source of Fe^2+^ ions for the Fenton reaction.^17,18^

There are several research studies on TNT treatment using the Fenton or Fenton-like process combining ultrasonic, UV, and the nZVI catalyst. Li *et al.*^19^ investigated the treatment efficiency of 2,4,6-trinitrotoluene using different oxidation processes, observing that the sono-Fenton process achieved the highest treatment efficiency of 87% of TNT after 30 min at pH 3, with a ratio of H_2_O_2_/Fe = 10 : 1, *C*^0^_TNT_ = 30 mg L^−1^, and *C*_Fe_ = 0.5 mM. Hashemi *et al.*^5^ showed that the TNT treatment efficiency at an initial concentration of 10 mg L^−1^, pH 3, with 2 mM Fe^2+^ ions, and 40 mM H_2_O_2_ by the sono-Fenton process could reach 100% after 20 min, while the efficiency of the photo-Fenton process was 97% after 60 min. Nguyen *et al.* also evaluated the efficiency of TNT treatment using a photo-Fenton process, and reported that the maximum TNT treatment efficiency reached 98.9% in 60 min under the conditions of *C*^0^_TNT_ = 49.58 mg L^−1^, H_2_O_2_/Fe^2+^ ratio of 20, pH 3.^20^ Next, the authors studied the effectiveness of yellow wastewater treatment using the photo-Fenton process, showing that the TNT treatment efficiency reached 97.70% at a TNT initial concentration of 84.58 mg L^−1^, pH 3, 7.19 mM ion Fe^2+^, and H_2_O_2_/Fe^2+^ ratio of 20.^21^ Ali Reza *et al.* researched TNT treatment with nZVI particles and reported achieving 91% efficiency at 30 mg per L TNT, pH 3.5, with 104.8 mg per L Fe^0^, and 675 mg per L H_2_O_2_.^22^ Marcio Barreto *et al.* studied the treatment of yellow wastewater containing TNT components using an nZVI pretreatment stage combined with the Fenton process, showing the system had the ability to treat 100% TNT, 87.5% total phenol, 95.4% COD.^23^ These research findings demonstrated the efficacy of ultrasound, light, Fenton, and nZVI catalyst, as well as their combination, for the treatment of TNT. Hence, the integration of the sono–photo-Fenton-like process with nZVI has potential as a novel and efficient approach for the elimination of persistent organic pollutants.

This study aimed to assess the efficacy of the sono–photo-Fenton-like system, utilizing zero-valent iron nanoparticles as a catalyst, for the treatment of 2,4,6-trinitrotoluene (TNT) in an aqueous solution. The objective of the project was to develop a cutting-edge solution that is highly efficient, minimizes the production of secondary waste, and enhances existing water-treatment processes.

## Results and discussion

### Characterizations

**Fig. 1:**
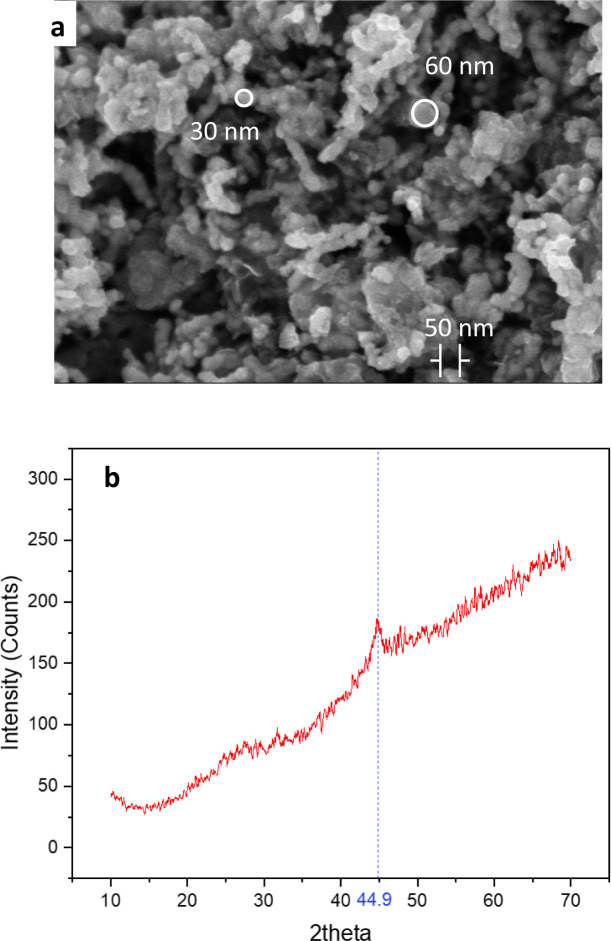
SEM image (a) and XRD spectra (b) of the synthesized iron nanoparticles.

Scanning electron microscopy (SEM), using a Hitachi S-4800 instrument from Japan, was employed to investigate the surface morphology and particle size of the produced materials. The data shown in Fig. 1a demonstrate that the synthesized particles were sphere-shaped with a mean particle size between 30 and 60 nm. This result was consistent with the TEM image of the nZVI nanoparticles, as shown in Fig. S1.† In addition, the crystal structure and phase of the produced nZVI material were analyzed using X-ray diffraction (XRD) with a Panalytical instrument from the Netherlands. The XRD examination revealed the distinctive peak of zero-valent iron at 2*θ* ≈ 44.9° (Fig. 1b).^24^

### Effect of the temperature

**Fig. 2:**
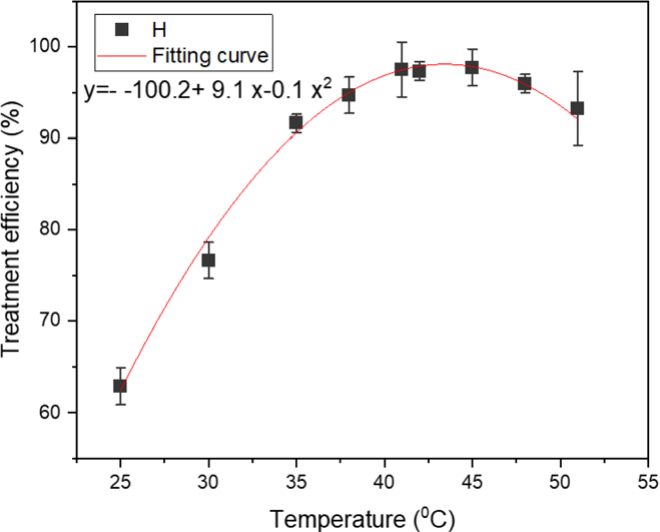
Effect of temperature on TNT treatment efficiency in aqueous solution with pH 3, *C*_H_2_O_2__ = 40 mM, *C*_Fe^0^_ = 2 mM, *C*^0^_TNT_ = 50 mg L^−1^, ultrasound = 40 kHz and 60 W, UV = 254 nm and 10 W, reaction time 30 min.

The effect of temperature on the efficacy of TNT treatment was examined utilizing a sono–photo-Fenton-like procedure, with temperatures ranging from 25 °C to 50 °C (see Fig. 2). The findings suggest that the effectiveness of the TNT treatment was greater within the temperature range of 40 °C to 45 °C. With the increase in temperature from 25 °C to 40 °C, the rate of TNT elimination increased from 62.88% to 97.53%. Nevertheless, as the temperature rose higher to 50 °C, the effectiveness of the TNT treatment decreased to 93.27%. The rise in temperature accelerated the reaction rate between H_2_O_2_ and Fe^2+^, resulting in a higher production of hydroxyl radicals.^25,26^ Wang *et al.* found that as the temperature of the solution increased, the development of cavitation bubbles under the impact of ultrasonic waves also increased. This led to the synthesis of more hydroxyl radicals, improving the removal effectiveness.^27^

Conversely, the rate of spontaneous breakdown of Fe^2+^ ions and H_2_O_2_ likewise escalated with the increase in solution temperature, resulting in a decline in the degradation efficiency. In addition, M. Haghmohammadi *et al.* reported that the distinct properties of wastewater and the dynamics of the reaction system are crucial elements in choosing the most favorable reaction temperature.^28^

### Effect of the nZVI catalyst dosage

**Fig. 3:**
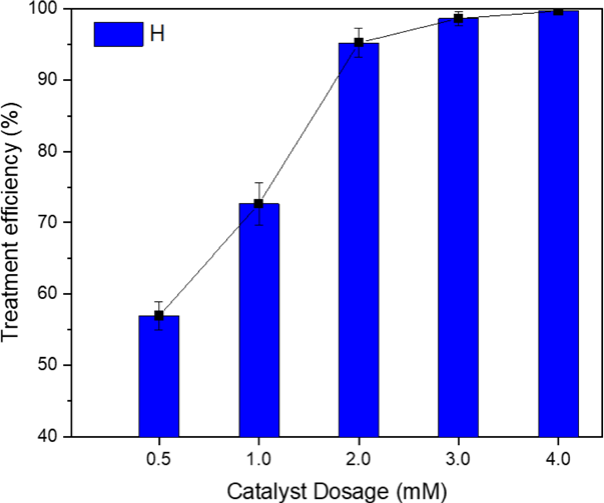
Impact of the dosage of zero-valent iron nanoparticles (nZVI) catalyst on the efficiency of TNT treatment (pH 3, *C*_H_2_O_2__ = 40 mM, *T* = 40 °C, *C*^0^_TNT_ = 50 mg L^−1^, ultrasound = 40 kHz and 60 W, UV = 254 nm and 10 W, reaction time = 30 min).

The effect of the amount of zero-valent iron nanoparticles on the effectiveness of TNT treatment is shown in Fig. 3. It was evident that as the dose of the catalyst was increased from 0.5 mM to 2 mM, the TNT removal efficiency was improved from 56.96% to 95.20%. When increasing the catalyst dose to 4 mM, the efficiency was able to reach an impressive 99.64%. Mina Hagh *et al.* found that raising the concentration of the heterogeneous catalyst carbon/Fe_3_O_4_ from 0.2 to 2 g L^−1^ improved the treatment efficiency for 4-chlorophenol in a sono-Fenton system, with an increase in efficiency from 69% to 85%.^28^ According to N. Thomas *et al.*, the use of ultrasonic irradiation improves the dispersion of iron nanoparticles in the solution, leading to an increase in treatment efficiency.^16^

In addition, a higher amount of catalyst leads to an elevated generation of cavitation bubbles on the catalyst surface and in the cavitation holes, resulting in higher concentrations of hydroxyl radicals.^29^ Nevertheless, as indicated by the research conducted by C. Lai *et al.*, the efficacy of pollutant removal may diminish when the catalyst concentrations are elevated, owing to the possible interaction between Fe^2+^ ions and hydroxyl radicals generated on the surface of the catalyst.^30^ Moreover, a higher catalyst dosage could lead to the dispersion of ultrasonic radiation and prevent light transmission through the solution.^31^

### Effect of the light wavelength

**Fig. 4:**
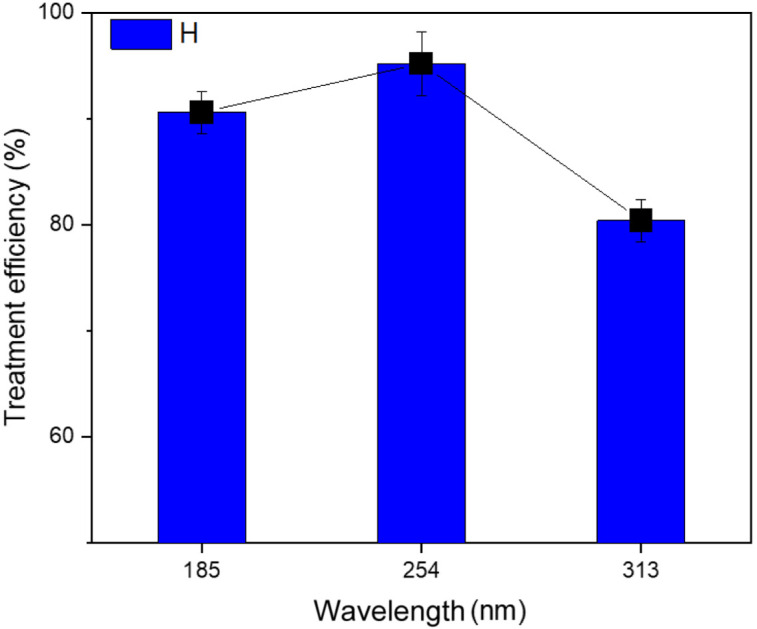
Effect of the light wavelength on the efficiency of TNT treatment (pH 3, *C*_H_2_O_2__ = 40 mM, *C*_Fe^0^_ = 2 mM, *T* = 40 °C, *C*TNT0 = 50 mg L^−1^, ultrasound = 40 kHz and 60 W, reaction time = 30 min).

The results indicate that the ultraviolet lamp with a wavelength of 254 nm achieved the best TNT treatment efficiency, reaching 95.2% (Fig. 4). The efficiency at a wavelength of 185 nm UV was 90.6%, while the efficiency at a wavelength of 313 nm UV was considerably lower, reaching only 80.4%. In H. Nguyen *et al.*'s investigation, the highest level of removal effectiveness was achieved using 254 nm UV light. In contrast, the removal efficiencies at 185 nm UV and 313 nm UV were comparable.^20^ As the wavelength decreases, the intensity of light increases.^32^ Thus, lights that emit wavelengths primarily in the UVC (200–275 nm) and UVD (100–200 nm) ranges are more likely to improve treatment effectiveness compared to lights in the UVA (320–420 nm) and UVB (275–320 nm) ranges. This is because they directly break down H_2_O_2_ into hydroxyl radicals and facilitate the formation of Fe^2+^ ions from iron complexes.^33^ Nevertheless, the intensity of UV radiation in water decreased as the wavelength became shorter. Hence, employing a UV lamp with shorter wavelengths may result in a reduction in the effectiveness of the treatment procedure, as demonstrated in the study conducted by Wang *et al.*^34^

### Effect of the ultrasound frequency

**Fig. 5:**
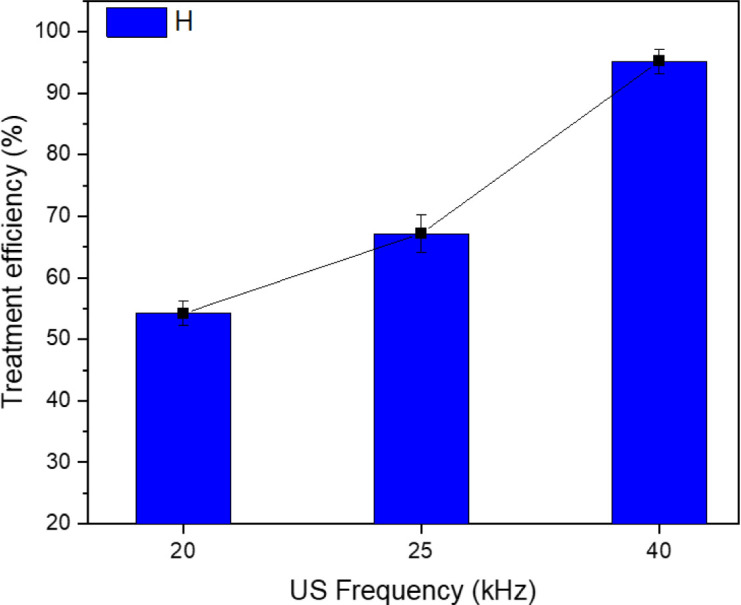
Effect of ultrasound frequency on the efficiency of TNT treatment. (pH 3, *C*_H_2_O_2__ = 40 mM, *C*_Fe^0^_ = 2 mM, *T* = 40 °C, *C*TNT0 = 50 mg L^−1^, UV = 254 nm and 10 W, reaction time = 30 min).

Fig. 5 shows that the efficacy of TNT therapy reached 95.2% under exposure to an ultrasonic frequency of 40 kHz. The efficiency at a frequency of 20 kHz was 54.2%, whereas at a frequency of 25 kHz it was 67.2%. Consequently, the effectiveness of TNT treatment was enhanced when the sono–photo-Fenton system was utilized in conjunction with zero-valent iron nanoparticles at elevated frequencies. Hoffmann *et al.* conducted a study that found that the effectiveness of TNT treatment with ultrasound at a frequency of 500 kHz was greater than that at 20 kHz.^35^ They also reported that low-frequency ultrasound improved the metabolic exchange in the response system.

Nevertheless, when the frequencies increases, the energy required for the formation of cavitation bubbles escalates, resulting in the generation of smaller bubbles with a greater concentration of energy and a quick release of hydroxyl radicals. T. Nguyen *et al.* examined the impacts of cavitation and chemical reactions across a range of ultrasonic frequencies, specifically from 22 kHz to 4880 kHz. The report conclusion states that when the frequency increases, both cavitation and chemical impacts are intensified.^36^

### Effect of the pH and H_2_O_2_/Fe^0^ ratio

**Fig. 6:**
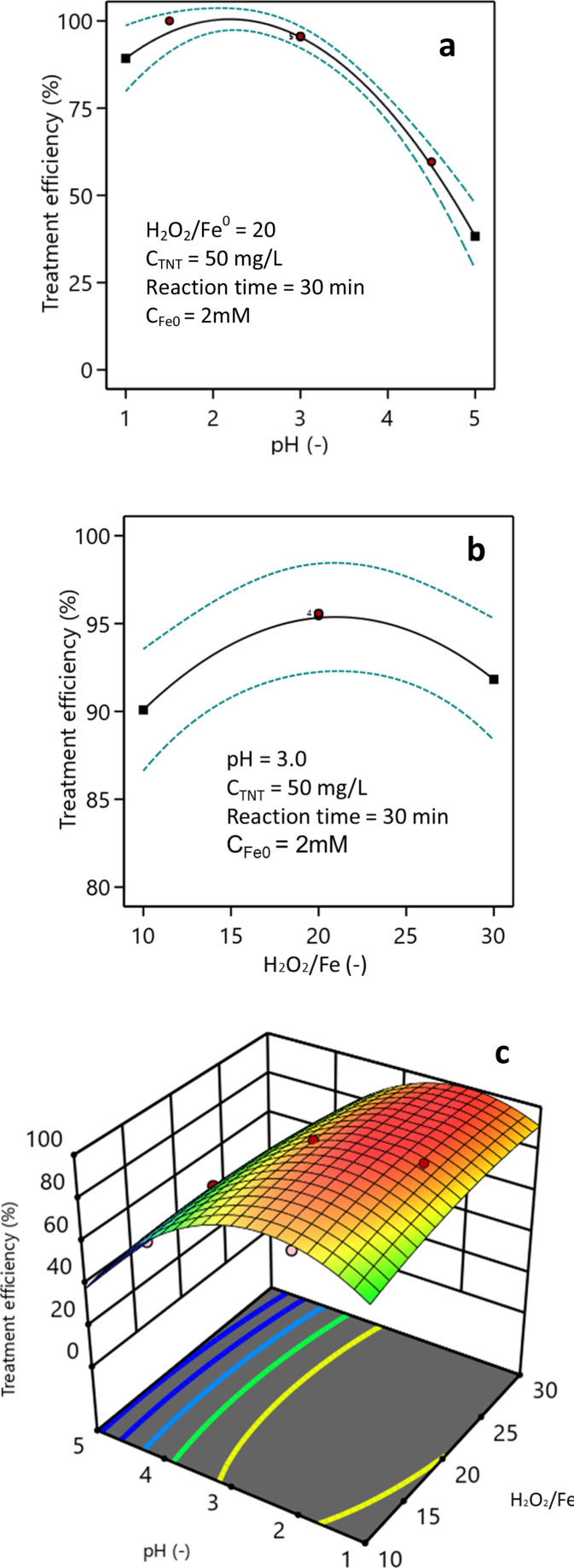
Effect of the H_2_O_2_/Fe^0^ ratio (a), pH (b) and their combination (c) on the efficiency of TNT treatment (*C*_Fe^0^_ = 2 mM, *C*^0^_TNT_ = 50 mg L^−1^, ultrasound = 40 kHz and 60 W, UV = 254 nm and 10 W, reaction time = 30 min).

Fig. 6a demonstrates the impact of pH on the efficacy of TNT treatment. Based on the findings obtained with a H_2_O_2_/Fe^0^ ratio of 20, it was evident that the most effective therapy for TNT could be accomplished at a pH level of 2–3. The treatment efficiency at pH 1 was 89.3%, which was lower than the efficiencies at pH 2 and pH 3 (98.7% and 95.2%, respectively). As the pH surpassed 3.0, the efficacy of TNT therapy progressively diminished, and the treatment efficacy for TNT fell dramatically from 74.7% at pH 4 to 38.3% at pH 5. Vaishnave and colleagues conducted research demonstrating that the sono–photo-Fenton system exhibited optimal efficacy for the treatment of Azure-B at a pH of 2.2.^37^ Lu *et al.* found that the presence of Fe_2_O_3_·H_2_O on the surface of heterogeneous catalysts at pH levels above 3, and their precipitation at pH levels of 5 and above, were the causes for the reduced effectiveness of the heterogeneous Fenton process.^38^ TNT decomposition efficiency decreases at higher pH values possibly due to the weaker oxidants, such as ferryl ions (*e.g.*, FeO^2+^), that are formed at higher pH values (pH ≥ 5) with higher selectivity and superiority in reactions with organic compounds compared to ˙OH (eqn (7)).^39^

7 Fe^2+^ + H_2_O_2_ → FeO^2+^ + H_2_O

Conversely, when the pH is less than 2.1, the presence of oxonium ions (H_3_O^2+^) and [Fe(H_2_O)_6_]^3+^ ions decreases the reactivity of Fe^2+^ ion and H_2_O_2_.^40,41^ When pH < 2, excess H^+^ will react with the free radical ˙OH according to eqn (8).^42^

8 ˙OH + H^+^ + e^−^ → O_2_H + H_2_O

Zhu *et al.*'s research indicates that the pH circumstances have an impact on the formation of hydroxyl radicals from cavitation bubbles caused by ultrasonic waves.^43^

Fig. 6b illustrates the impact of the H_2_O_2_/Fe^0^ ratio on the efficiency of TNT treatment. According to the research findings, increasing the H_2_O_2_/Fe^0^ ratio from 10 to 20 resulted in an improvement in the efficiency of TNT treatment. Specifically, the treatment efficiency increased from 90.1% to 95.2% when the reaction period was 30 min and the pH was 3. Nevertheless, as the H_2_O_2_/Fe^0^ ratio steadily rose from 25 to 30, the effectiveness of TNT treatment declined to 94.6% and 91.8%, respectively. As stated by Mina Hagh *et al.*, the ideal amount of H_2_O_2_ in the heterogeneous Fenton system is influenced by the concentration of heterogeneous catalysts, the pH, and temperature.^28^ The drop that can be observed in the TNT treatment efficacy is caused by the reaction between H_2_O_2_ and hydroxyl radicals, leading to a reduction in oxidation radicals, subsequently resulting in a drop in treatment efficacy.

Fig. 6c illustrates the influence of pH and the H_2_O_2_/Fe^0^ ratio on TNT treatment efficiency. The research findings indicate that the optimal treatment efficiency could be achieved at pH 2–3 and a H_2_O_2_/Fe^0^ ratio of 20. The equation representing the effect of pH (*X*_1_) and the H_2_O_2_/Fe^0^ ratio (*X*_2_) on treatment efficiency (*H*) is shown below as eqn (9):

9 % *H* = 26.67 + 40.14*X*_1_ + 2.67*X*_2_ − 0.28*X*_1_*X*_2_ − 7.89*X*_1_^2^ − 0.044*X*_2_^2^

By ANOVA analysis of the regression equation, *F*-value was 46.93, and the *P*-value was <0.0001. Additionally, according to Fit Statistics, the predicted, adjusted *R*-squared, and *R*^2^ values were 0.79, 0.95, and 0.97, respectively. Therefore, it could be concluded that the regression equation was statistically significant.

### Effect of the initial TNT concentration and reaction time

**Fig. 7:**
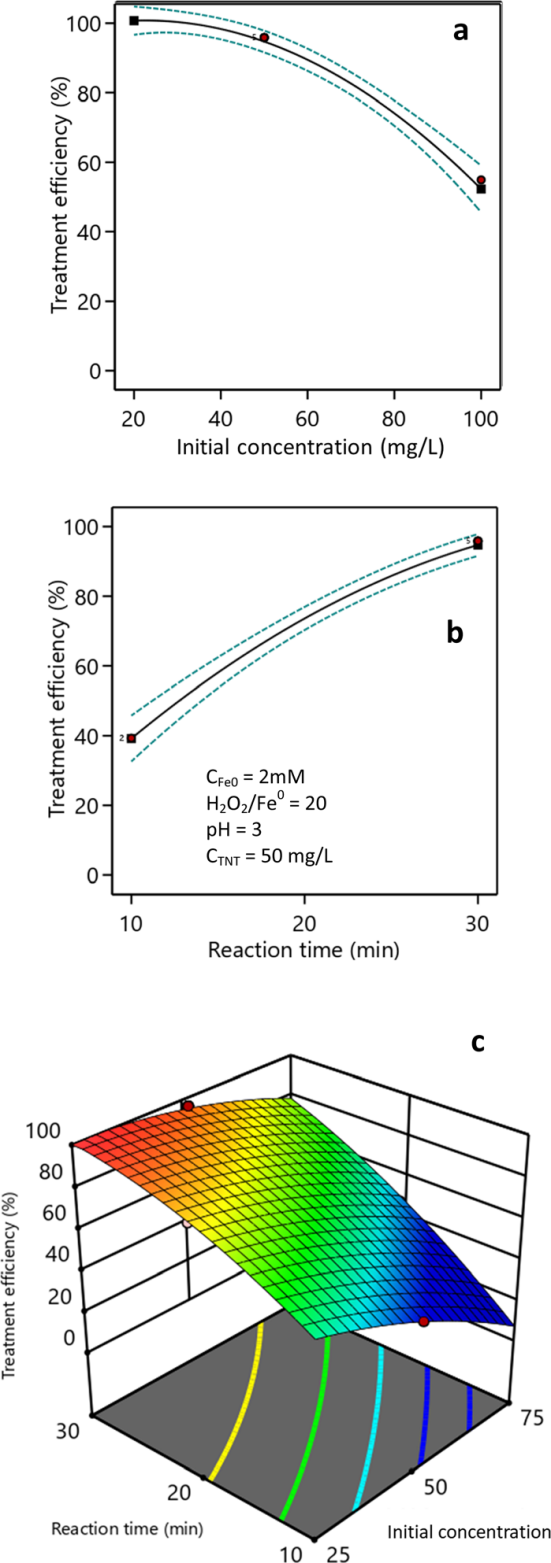
Effect of the initial TNT concentration (a), reaction time (b), and their combination (c) on TNT treatment efficiency (pH 3, *C*_H_2_O_2__ = 40 mM, *C*_Fe^0^_ = 2 mM, ultrasound = 40 kHz and 60 W, UV = 254 nm and 10 W).

The impact of the starting TNT concentration on the treatment efficiency is presented in Fig. 7a. The research findings suggest that when the pH was set to 3, the starting concentration of H_2_O_2_ was 40 mM, and the dosage of nZVI catalyst was 2 mM, the effectiveness of TNT treatment was reduced when the initial concentration of TNT in the solution was increased. When the initial TNT concentration was 25 mg L^−1^, the treatment efficiency reached 100% within 30 min reaction time. However, at initial TNT concentrations of 50, 75, and 100 mg L^−1^, the treatment efficiencies were 95.2%, 78.6%, and 52.3%, respectively. The decrease in treatment efficacy as the initial TNT concentration increased may have been due to the blocking of light transmission by the solution at higher concentrations. The color index of the solution increased from 200 Pt–Co to 800 Pt–Co when the initial TNT concentration was increased from 25 mg L^−1^ to 100 mg L^−1^. Vaishnave *et al.* Also observed a reduction in the effectiveness of Azure-B treatment in the sono–photo-Fenton process as the concentration of pollutants increased.^37^ Also, Hosseini *et al.* showed that elevated levels of pollutants could impede the penetration of UV irradiation to the catalyst surface, hence diminishing the effectiveness of hydroxyl radical generation.^44^ In addition, TNT molecules adsorbed on nZVI surface could hinder the approach of H_2_O_2_, leading to the generation of fewer hydroxyl free radicals on the nZVI surface and therefore a reduced removal rate.^39^

Fig. 7b demonstrates the influence of the reaction time on the effectiveness of treatment. The study demonstrated that, with an initial TNT concentration of 50 mg L^−1^, the efficiency of TNT treatment increased from 17.0% to 95.2% as the response time was extended from 5 min to 30 min. After a duration of 30 min, the effectiveness of the TNT treatment continued to improve, albeit not substantially.

Fig. 7c illustrates the correlation between the time it took for the reaction to occur and the starting concentration of TNT. The research findings indicated a positive correlation between the reaction time and the efficiency of TNT therapy. Greater starting TNT concentrations needed extended treatment durations to attain equivalent therapy efficacy compared to lower amounts. The correlation between the starting TNT concentration (*X*_3_) and reaction time (*X*_4_) on the treatment efficiency (*H*) is shown by eqn (10). The *F*-value was 113.48 and the *P*-value was less than 0.0001. The expected, adjusted *R*-squared, and *R*^2^ values were 0.89, 0.98, and 0.99, correspondingly.

10 % *H* = 40.71 − 0.58*X*_3_ + 3.85*X*_4_ + 0.031*X*_3_*X*_4_ − 0.008*X*_3_^2^ − 0.066*X*_4_^2^

### Influence of the ultrasonic power and light power

**Fig. 8:**
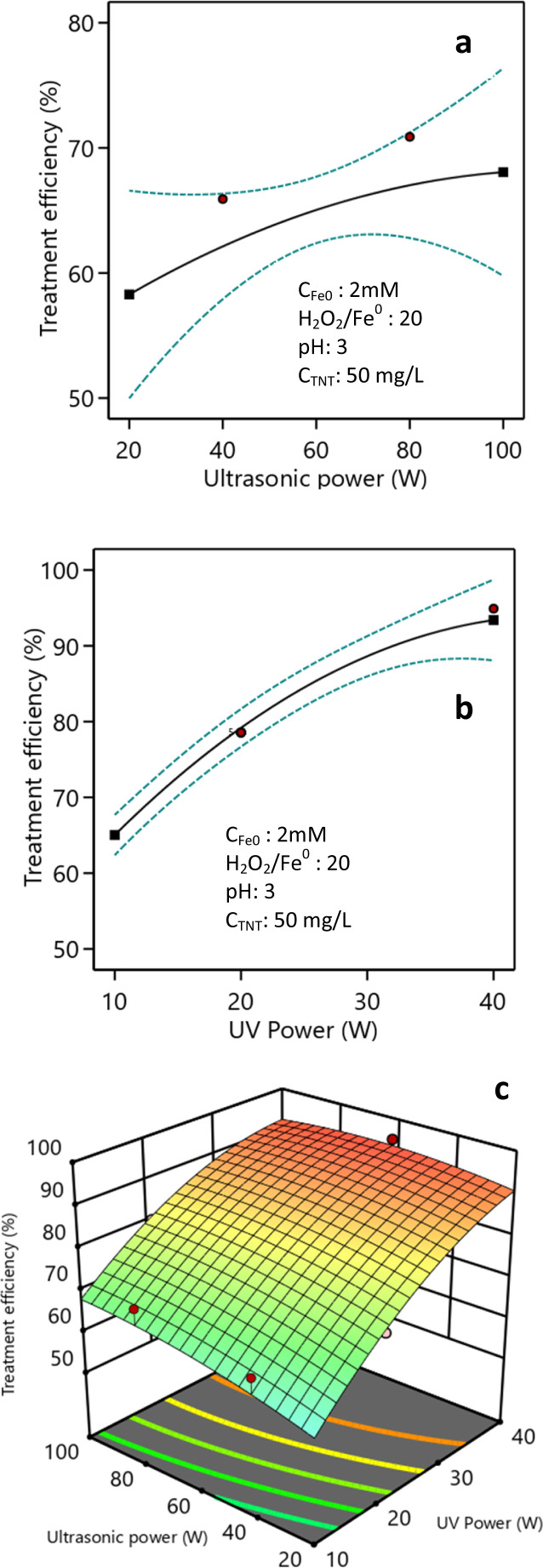
Impact of ultrasonic power (a) and light power (b) and their combination (c) (pH 3, *C*_H_2_O_2__ = 40 mM, *C*_Fe^0^_ = 2 mM, *C*^0^_TNT_ = 50 mg L^−1^, ultrasound = 40 kHz, UV = 254 nm, reaction time = 20 min).

The investigation into the impact of the ultrasonic power demonstrated that, following a 20 min response, escalating the ultrasonic power from 20 W to 80 W resulted in an increase in the TNT treatment efficiency from 73.8% to 80.4% (as depicted in Fig. 8a). Nevertheless, when the ultrasonic power was further raised to 90 W and 100 W, the efficiency of the TNT treatment showed only a minimal improvement, oscillating between 80.0% and 81.0%. Augmenting the ultrasonic power amplifies the generation of cavitation bubbles. At increased ultrasonic power, the bubbles rapidly attain their maximum size and undergo a sudden and forceful expansion, resulting in the production of hydroxyl radicals. This occurs due to the thermal breakdown of water molecules caused by the combination of high pressure and high temperature.^45,46^ The increase in capacity causes a shorter lifetime of the bubbles and the collapse of the bubbles occurs more quickly, leading to a greater production of ˙H and ˙OH free radicals, increasing their ability to attack the molecules.^47,48^

Increasing the ultrasonic power additionally enhanced the mass transfer and further supported dispersing the catalyst in the sono–photo-Fenton-like process.^29,49^ Additionally, the ultrasonic power affected the surface cleaning process of the catalyst, thereby enhancing the treatment efficiency.^50^ Conversely, elevating the ultrasonic power could impact the temperature of the solution. If the temperature exceeds the recommended range, it can have a detrimental impact on the treatment efficiency.

The study findings depicted in Fig. 8b indicate that the effectiveness of the TNT therapy was increased from 65.0% to 93.4% when the light intensity was increased from 10*W*/*L* to 40*W*/*L*. Increasing the light intensity promotes the generation of hydroxyl radicals on the catalyst's surface, leading to enhanced treatment effectiveness. Chen *et al.* found that increasing the intensity of light improves photochemical processes, leading to a greater effectiveness in treatment.^51^

Fig. 8c demonstrates the collective influence of the light intensity and ultrasonic power on the effectiveness of TNT treatment. It is evident from the figure that the increase in both ultrasonic power and light intensity led to a corresponding rise in the efficiency of TNT treatment. The impact of the ultrasonic power (*X*_5_) and light intensity (*X*_6_) on the effectiveness of treatment *H* is shown in eqn (11). The *F*-value was 113.48 and the *P*-value was less than 0.0001. The anticipated, adjusted *R*-squared, and *R*^2^ values, were 0.78, 0.96, and 0.98, respectively:

11 % *H* = 32.58 + 0.29*X*_5_ + 2.32*X*_6_ − 0.0035*X*_5_*X*_6_ − 0.0012*X*_5_^2^ − 0.023*X*_6_^2^

### Comparing the treatment efficiency of TNT using different advanced oxidation processes

**Fig. 9:**
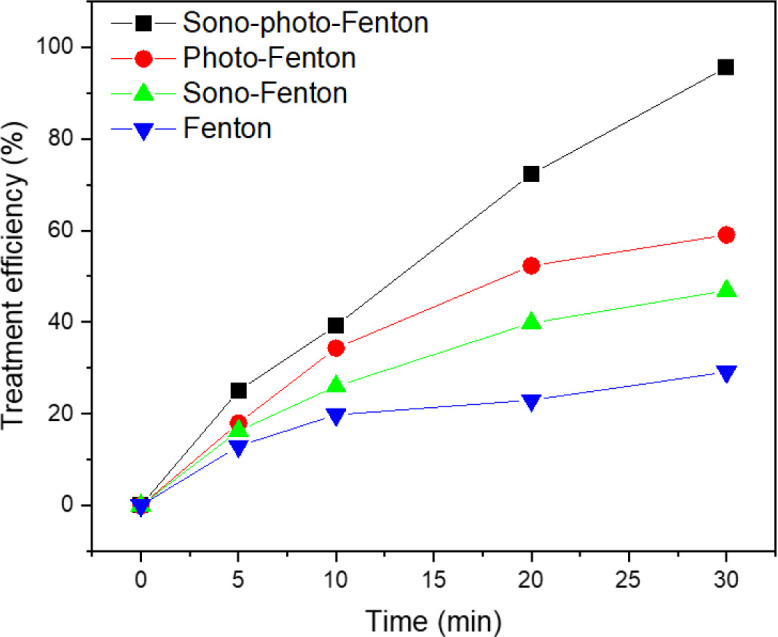
Comparison of the efficiency of TNT treatment among various advanced oxidation methods (pH 3, *C*_H_2_O_2__ = 40 mM, *C*_Fe^0^_ = 2 mM, *C*^0^_TNT_ = 50 mg L^−1^, reaction time = 30 min).

The research findings indicate that the most effective method for treating TNT is by combining the heterogeneous sono–photo-Fenton system with a zero-valent iron catalyst, resulting in a 95.2% efficiency (Fig. 9). The combination of the photo-Fenton-like process and zero-valent iron yielded an efficiency of 59.02%, while the efficiencies of the sono-Fenton-like and Fenton-like processes were 43.73% and 30.2%, respectively. Based on these findings, it could be deduced that the sono–photo-Fenton system paired with a zero-valent iron catalyst demonstrated the maximum efficiency in treating TNT. Thus, the combined use of ultrasound and light can greatly improve the effectiveness of the heterogeneous Fenton process using zero-valent iron.

Compared to the homogeneous sono-Fenton process and photo-Fenton processes (Table 1), it can be seen that the removal efficiency of TNT by the sono–photo-Fenton process combined with nZVI was higher under the same time and initial conditions. Furthermore, the utilization of heterogeneous catalysts significantly decreases the quantity of sludge produced in comparison to homogeneous Fenton methods, owing to the catalyst's capacity to be retrieved and used again. Next, there is a consideration of the costs and the mitigation of secondary pollution.

**Table 1:** TNT removal efficiency by the sono-Fenton and photo-Fenton processes

Process type	*C* ^0^_TNT_ (mg L^−1^)	pH	Reaction time (min)	Reaction conditions	Removal efficiency	Ref.
Sono-Fenton	30	3	30	*C*_H_2_O_2__ = 5 mM; *C*_Fe(II)_ = 0.5 mM	83%	19
Photo-Fenton	50	3	30	*C*_H_2_O_2__ = 40 mM; *C*_Fe(II)_ = 2 mM	70%	5
Sono-Fenton	50	3	30	*C*_H_2_O_2__ = 40 mM; *C*_Fe(II)_ = 2 mM	90%	5
Photo-Fenton	49.58	3	60	*C*_H_2_O_2__ = 35 mM; *C*_Fe(II)_ = 1.75 mM	98.9%	20

### Reusability of the nZVI catalyst

**Fig. 10:**
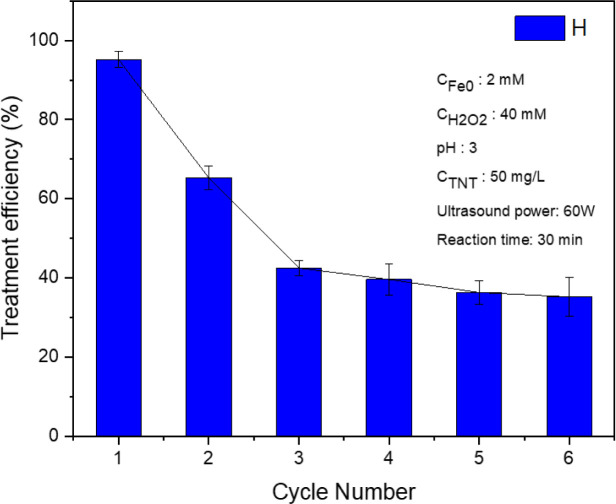
TNT treatment efficiency of the reused nZVI catalyst in the sono–photo-Fenton-like process (pH 3, *C*_H_2_O_2__ = 40 mM, *C*_Fe^0^_ = 2 mM, *C*^0^_TNT_ = 50 mg L^−1^, ultrasonic = 40 kHz and 60 W, UV = 254 nm and 10 W, reaction time = 30 min).

The micro iron catalyst was retrieved following each reaction through the utilization of a magnet, and subsequently subjected to multiple washes with distilled water and 99% alcohol, and finally dehydrated in a vacuum desiccator. According to the experimental findings, the effectiveness of TNT therapy declined progressively from 95.2% during the initial reuse to 65.3% during the second reuse, and further dropped to 42.5% during the third cycle (as shown in Fig. 10). The drop in efficiency was caused by the production of Fe_2_O_3_ oxide on the surface of the nano iron during the reaction, which obstructs the involvement of nZVI in the reaction. Moreover, the presence of Fe^0^ diminished as a result of its reaction within the Fenton system, hence lowering the effectiveness of the heterogeneous catalyst. Furthermore, while undergoing the treatment, iron particles were released into the solution in the form of Fe^2+^ ions.

During the third to sixth cycles, the efficacy of TNT treatment gradually declined from 45.2% to 35.2% in the sixth cycle. The SEM image and XRD pattern of nZVI after treatment were obtained and are shown in Fig. S2.† The results show that the used nZVI nanoparticles had negligible change in their morphology and crystallinity in comparison to the material before treatment.

The proposed degradable mechanism of TNT can be described in two stages (Fig. S3†). In the first stage, the degradation of TNT by nZVI can occur through reduction reactions, converting the nitro groups into amino groups.^52^ The most easily observed reduction product of TNT by nZVI is triaminetoluene (TAT), which has lower biological toxicity.^3^ The hydroxyl radical (˙OH) generated from the SPF process abstracts a hydrogen atom from the methyl group, creating a methyl radical TAT, which then oxidizes to form 2,4,6-triaminobenzoic acid. This is followed by aromatic acid decarboxylation, as reported by Liou *et al.*^53^ Finally, hydrolysis and mineralization lead to products such as CO_2_ and H_2_O. In the second way, TAT can be converted into another substance through the cleavage of the amine group and the addition of the hydroxyl radical to the aminoaromatic.^19^ Subsequently, the methyl group is attacked by ˙HO, leading to transformation into 2,4,6-trihydroxybenzoic acid. Finally, the ring-opening oxidation reaction results in the formation of organic acid compounds, CO_2_, and H_2_O.

## Conclusions

The experimental results when combining the sono–photo-Fenton-like process with zero-valent iron nanoparticles show that the optimal temperature for the reaction was 40–45 °C. Under the optimal temperature conditions, increasing the concentration of the heterogeneous catalyst from 0.5 mM to 4 mM enhanced the efficiency of TNT treatment. The highest efficiency for the removal of TNT in aqueous media was achieved at pH 2–3 and an H_2_O_2_/Fe^0^ ratio of 20. The initial concentration of TNT also affects the treatment efficiency. Higher initial concentrations required longer reaction times. Evaluation of the impact of ultrasound power and light intensity showed that treatment efficiency increased with increasing the ultrasound power and light intensity. Additionally, the optimal wavelength for the experiment was 254 nm, while increasing the frequency of ultrasound enhanced the treatment efficiency. The research results also indicate that the efficiency of TNT treatment using the sono–photo-Fenton-like process combined with zero-valent iron catalyst was significantly higher than that of the photo-Fenton-like, sono-Fenton-like, and Fenton-like processes under the same conditions. However, it is necessary to continue with studies on the recovery and effective reuse of nano catalysts in order to apply them to pilot scales.

## Experimental section

### Chemicals and instrumentation

The key materials and reagents were: 2,4,6-trinitrotoluene (95%, Vietnam), Fe_2_SO_4_·7H_2_O, FeCl_3_·6H_2_O, CuCl_2_·2H_2_O (99%, Xilong Scientific, China), NaBH_4_, NaOH, H_2_SO_4_ 98% (Xilong Scientific, China), H_2_O_2_ (30%, Analytical Reagent, China), de-ionized water (Milli Q).

**Fig. 11:**
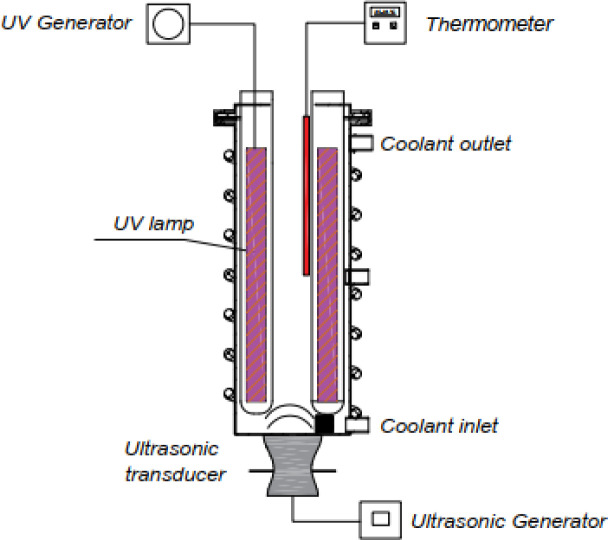
Design of the experimental reactor for the sono–photo-Fenton process.

The experimental reactor for investigating the effectiveness of the sono–photo-Fenton is shown in Fig. 11. The cylinder reactor with a 1 L capacity was made of stainless steel (SUS 304) with a tapered bottom to enhance the ultrasound wave transmission efficiency. An ultrasonic transducer was attached to the bottom of the reactor and connected to an ultrasonic generator (with variable control of the frequency from 20 to 50 kHz and power from 0 to 300 W, Sonigreen, Vietnam). A total of 4 UV lamps could be placed in the axial position inside the reactor to evaluate different UV power levels. The temperature inside the reactor could be controlled through the temperature sensor and cooling system around the reactor.

### Synthesis of the nano zero-valent iron

Zero-valent iron nanoparticles were synthesized using a facile chemical reduction method under atmospheric conditions.^24^ First, 0.5406 g FeCl_3_·6H_2_O and 50 mL deionized (DI) water were added to a 100 mL glass beaker and stirred at room temperature until completely dissolved. Next, iron chloride solution was added into a 500 mL three-neck round bottom flask and constantly stirred using a magnetic stirrer. Next, 100 mL of NaBH_4_ 0.1 M solution was titrated slowly into the flask. After adding the reducing agent, this mixture solution was continuously stirred for 2 h. The obtained black-colored particles were filtered from the liquid phase through a Buchner funnel by vacuum suction. The solid black product was washed several times using absolute ethanol (99%). Finally, the synthesized particles were dried in vacuum using a desiccator for 24 h.

### Treatment efficiency experiments

The effects of each factor, including temperature (ranging from 25 °C to 50 °C), nZVI catalyst concentration (ranging from 0.5 mM to 4 mM), ultrasound frequency (20, 25, 40 kHz), and light wavelength (185, 254, 313 nm) on the TNT treatment efficiency by the sono–photo-Fenton-like process were evaluated under the initial conditions: *C*^0^_TNT_ = 50.00 mg L^−1^, *C*_H_2_O_2__ = 40 mM, UV lamp power = 10 W, ultrasound power = 60 W, and pH 3.

In addition, the effects of pH (1–5) and H_2_O_2_/Fe^0^ ratio (5–35); initial TNT concentration (0–100 mg L^−1^), and reaction time (10–50 min); ultrasound power (20–100 W) and UV power (10–40 W) were studied using Design Expert 13 software through the Response Surface Methodology (RSM) with a 5-point central composite design (CCD). The model and regression equations were validated for experimental fit using an ANOVA test (with a significance level of *P*-value = 0.05).

Under the optimal conditions of pH 3, *C*_H_2_O_2__ = 40 mM, *C*_Fe^0^_ = 2 mM, *C*^0^_TNT_ = 50 mg L^−1^, and reaction time of 30 min, experiments were carried out to compare the TNT removal efficiency of the sono–photo-Fenton-like, photo-Fenton-like, sono-Fenton-like, and Fenton-like methods combined with nZVI.

Finally, experiments were conducted reusing the recovered nZVI catalyst under the same conditions, *i.e.*, pH 3, *C*_H_2_O_2__ = 40 mM, *C*_Fe^0^_ = 2 mM, *C*^0^_TNT_ = 50 mg L^−1^, ultrasound frequency = 40 kHz and power = 60 W, UV wavelength = 254 nm and power = 10 W, and reaction time of 30 min.

### Analytical methods

The TNT concentration in the solution was analyzed using a high-performance liquid chromatography (HPLC) system (Agilent, USA, 1100 Series) with a Hypersil C18 column (200 × 4 mm), and mobile phase consisting of acetonitrile and water at a ratio of 65/35, operating at a pressure of 280 bar, and pH 7.

The TNT treatment efficiency for each sample was calculated following eqn (12):12
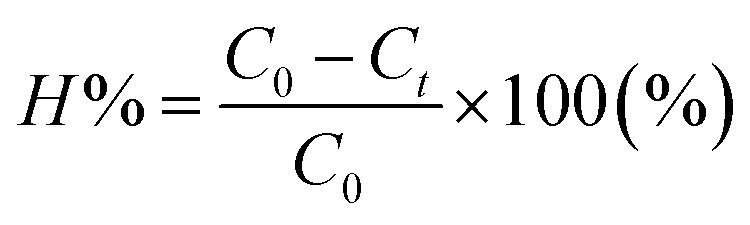
where *H* is the treatment efficiency, and *C*_o_ and *C*_*t*_ are the concentrations of TNT at the initial time and at time *t*, respectively.

## Data availability

Data for this article, including SEM, XRD, TEM, and adsorption analyses, are available at Open Science Framework at https://osf.io/me67s.

## Author contributions

VHN, STP: investigation, data collection, writing-original draft preparation. VNT: resources, reviewing and editing; VHN, STP: writing-reviewing and editing. DDL: visualization, editing, funding acquisition & supervision. All authors approved the manuscript.

## Conflicts of interest

The authors declare that they have no known competing financial interests or personal relationships that could have appeared to influence the work reported in this paper.

## Supplementary Material

RA-014-D4RA03907F-s001

## References

[cit1] Ayoub K., van Hullebusch E. D., Cassir M., Bermond A. (2010). J. Hazard. Mater..

[cit2] Ribeiro E., Silva F., Paiva T. (2012). J. Environ. Sci. Health, Part A: Toxic/Hazard. Subst. Environ. Eng..

[cit3] Barreto-Rodrigues M., Silva F. T., Paiva T. C. B. (2009). J. Hazard. Mater..

[cit4] Li J., Zhou Q., Li M., Liu Y., Song Q. (2021). J. Environ. Sci..

[cit5] Hashemi M., Sagharlo N. (2020). J. Adv. Environ. Health Res..

[cit6] Moradi M., Elahinia A., Vasseghian Y., Dragoi E.-N., Omidi F., Mousavi Khaneghah A. (2020). J. Environ. Chem. Eng..

[cit7] Dükkancı M. (2018). Ultrason. Sonochem..

[cit8] Moradi S., Rodriguez-Seco C., Hayati F., Ma D. (2023). ACS Nanosci. Au.

[cit9] ElShafei G. M. S., Yehia F. Z., Eshaq G., ElMetwally A. E. (2017). Sep. Purif. Technol..

[cit10] Shokri A. (2018). Int. J. Ind. Chem..

[cit11] Gogate P., Manickam S. (2004). Sep. Purif. Technol..

[cit12] Ferkous H., Hamdaoui O., Merouani S. (2015). Ultrason. Sonochem..

[cit13] Zhou M., Yang H., Xian T., Li R. S., Zhang H. M., Wang X. X. (2015). J. Hazard. Mater..

[cit14] Khataee A., Gholami P., Vahid B., Joo S. W. (2016). Ultrason. Sonochem..

[cit15] Hussain S., Aneggi E., Goi D. (2021). Environ. Chem. Lett..

[cit16] Thomas N., Dionysiou D. D., Pillai S. C. (2021). J. Hazard. Mater..

[cit17] Akanbi O., Kim I., Cha D., Attavane A., Hubbard B., Chiu P. (2022). Propellants, Explos., Pyrotech..

[cit18] Liu J., Ou C., Han W., Faheem D., Shen J., Bi H., Sun X., Li J., Wang L.-J. (2015). RSC Adv..

[cit19] Li Y., Zhang W., Mu K., Li S., Wang J., Zhang S., Wang L. (2023). Int. J. Environ. Res. Public Health.

[cit20] Nguyen H., Pham Son T., Le Minh T., Nguyen Le Tu Q. (2022). J. Mil. Sci. Tech..

[cit21] Nguyen V. H., Pham S. T., Le M. T., Le T. D. (2023). Journal of Military Science and Technology.

[cit22] Zarei A. R., Rezaeivahidian H., Mehrabi G. R., Farajpour T. (2019). Environ. Prog. Sustainable Energy.

[cit23] Barreto-Rodrigues M., Silva F. T., Paiva T. C. B. (2009). J. Hazard. Mater..

[cit24] Boonruam P., Soisuwan S., Wattanachai P., Morillas H., Upasen S. (2020). ASEAN Eng. J..

[cit25] Bao T., Jin J., Damtie M. M., Wu K., Yu Z. M., Wang L., Chen J., Zhang Y., Frost R. L. (2019). J. Saudi Chem. Soc..

[cit26] Moreira F. C., Boaventura R. A. R., Brillas E., Vilar V. J. P. (2015). Appl. Catal., B.

[cit27] Wang C., Shih Y. (2015). Sep. Purif. Technol..

[cit28] Haghmohammadi M., Sajjadi N., Beni A. A., Hakimzadeh S. M., Nezarat A., Asl S. D. (2023). J. Water Process Eng..

[cit29] Zhong X., Royer S., Zhang H., Huang Q., Xiang L., Valange S., Barrault J. (2011). Sep. Purif. Technol..

[cit30] Lai C., Huang F., Zeng G., Huang D., Qin L., Cheng M., Zhang C., Li B., Yi H., Liu S., Li L., Chen L. (2019). Chemosphere.

[cit31] ElMetwally A. E., Eshaq G. h., Al-Sabagh A., Yehia F. Z., Philip C. A., Moussa N., Elshafei G. (2019). Sep. Purif. Technol..

[cit32] Matafonova G., Batoev V. (2018). Water Res..

[cit33] Guo W., Li T., Chen Q., Wan J., Zhang J., Wu B., Wang Y. (2021). Chemosphere.

[cit34] Wang Y., Sun Y., Wang Z., Kong Q., Wu B., Wan J., Guo W. (2024). Environ. Technol..

[cit35] Hoffmann M. R., Hua I., Höchemer R. (1996). Ultrason. Sonochem..

[cit36] Thanh Nguyen T., Asakura Y., Koda S., Yasuda K. (2017). Ultrason. Sonochem..

[cit37] Vaishnave P., Kumar A., Ameta R., Punjabi P. B., Ameta S. C. (2014). Arabian J. Chem..

[cit38] Lu J., Zhou Y., Ling L., Zhou Y. (2022). Chem. Eng. J..

[cit39] Wang L., Yang J., Li Y., Lv J., Zou J. (2016). Chem. Eng. J..

[cit40] Monteagudo J. M., Durán A., Martín I. S., García S. (2014). Appl. Catal., B.

[cit41] Zhou Q., Liu Y., Yu G., He F., Chen K., Xiao D., Zhao X., Feng Y., Li J. (2017). Polym. Degrad. Stab..

[cit42] Sun J.-H., Sun S.-P., Sun J.-Y., Sun R.-X., Qiao L.-P., Guo H.-Q., Fan M.-H. (2007). Ultrason. Sonochem..

[cit43] Zhu Y., Fan W., Feng W., Wang Y., Liu S., Dong Z., Li X. (2021). J. Hazard. Mater..

[cit44] Hosseini M., Kahkha M. R. R., Fakhri A., Tahami S., Lariche M. J. (2018). J. Photochem. Photobiol., B.

[cit45] Khataee A., Kayan B., Gholami P., Kalderis D., Akay S. (2017). Ultrason. Sonochem..

[cit46] Camargo-Perea A. L., Rubio-Clemente A., Peñuela G. A. (2020). Water.

[cit47] Hou L., Wang L., Royer S., Zhang H. (2016). J. Hazard. Mater..

[cit48] Ferkous H., Merouani S., Hamdaoui O., Pétrier C. (2017). Ultrason. Sonochem..

[cit49] Dükkancı M. (2019). Water Sci. Technol..

[cit50] Khan M. A. N., Siddique M., Wahid F., Khan R. (2015). Ultrason. Sonochem..

[cit51] Chen Y., Lu A., Li Y., Yip H. Y., An T., Li G., Jin P., Wong P.-K. (2011). Chemosphere.

[cit52] Mdlovu N. V., Lin K.-S., Hsien M.-J., Chang C.-J., Kunene S. C. (2020). J. Taiwan Inst. Chem. Eng..

[cit53] Liou M.-J., Lu M.-C., Chen J.-N. (2003). Water Res..

