# Supplementary material for: Effective treatment of 2,4,6-trinitrotoluene from aqueous media using a sono–photo-Fenton-like process with a zero-valent iron nanoparticle (nZVI) catalyst
Source: RSC Adv. 2024 Jul 29;14(33):23720–9. doi: 10.1039/d4ra03907f (PMC11284922; doi:10.1039/d4ra03907f)
Supplement: RA-014-D4RA03907F-s001 [file RA-014-D4RA03907F-s001.pdf]

Supplementary Information (SI) for RSC Advances.
This journal is © The Royal Society of Chemistry 2024

Supporting Information

Effective treatment of 2,4,6-Trinitrotoluen from aqueous media using Sono-Photo-Fenton-like process with zero-valent iron nanoparticles (nZVI) catalyst

Hoang Van Nguyen[a], Son Tung Pham[a,*], Toan Ngoc Vu[a], Huong Van Nguyen[a], Duong Duc La[b,*]

[a] *Institute of New Technology, Hanoi, Vietnam.*

[b] *Institute of Chemistry and Materials, Hanoi, Vietnam.*

** Corresponding authors: duc.duong.la@gmail.com, sontung231@gmail.com*

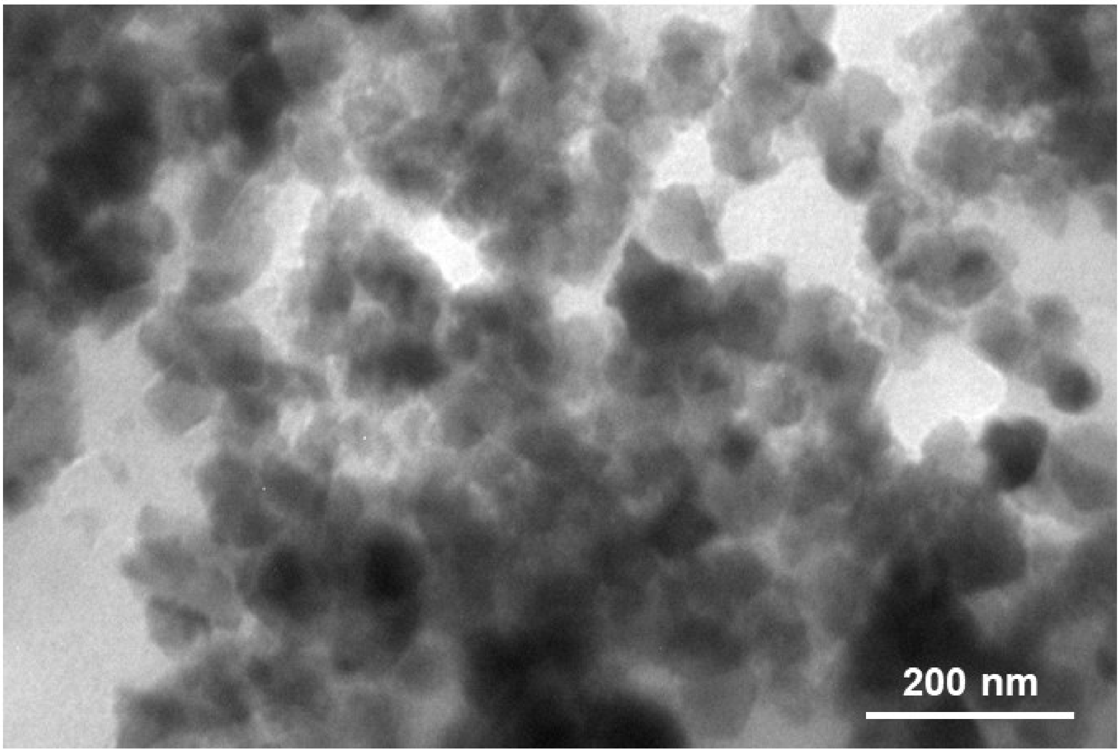

Figure S1. TEM image of nZVI nanoparticle

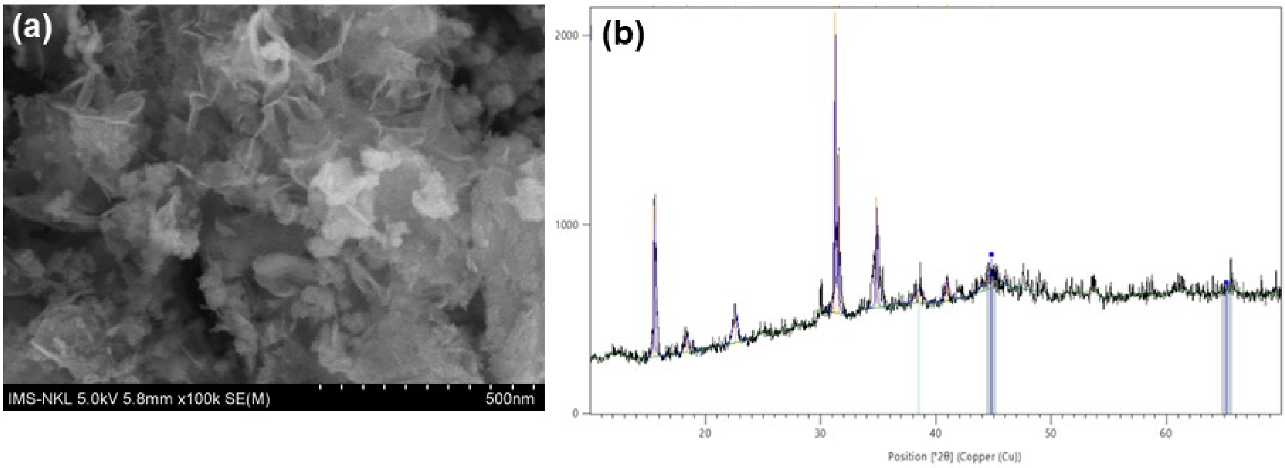

Figure S2. SEM image (a) and XRD pattern (b) of nZVI nanoparticles after treatment.

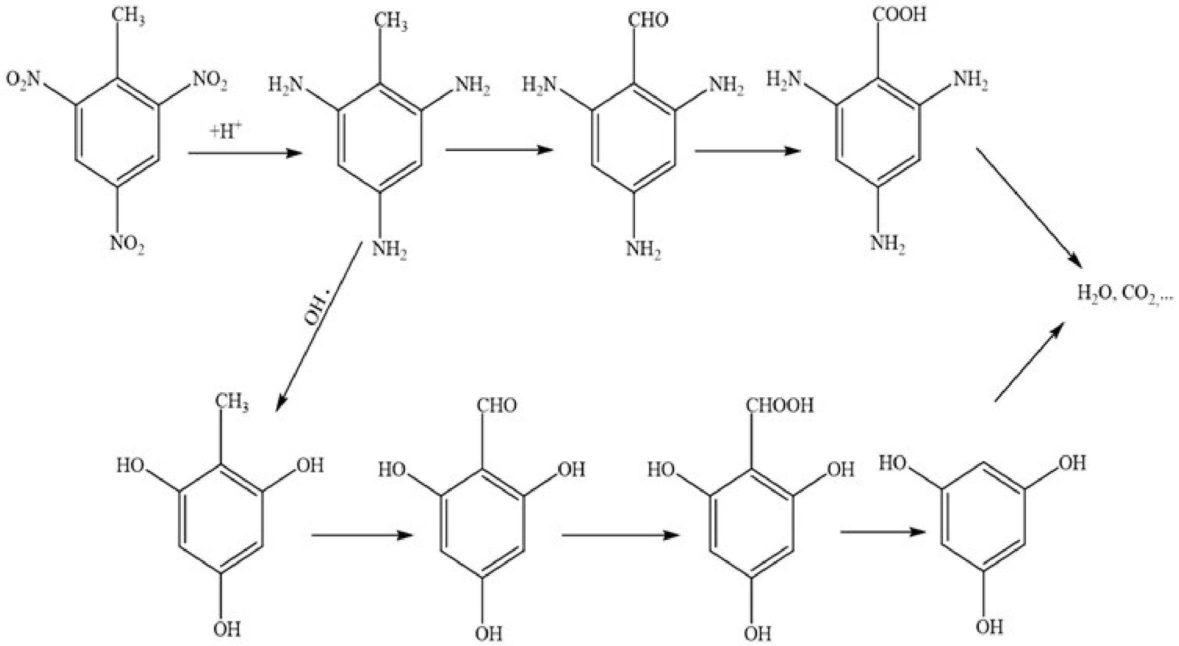

Figure S3. Proposed TNT degradation mechanismen by sono-photo-fenton process combined with nZVI
